# Vessel anatomy of urban *Celtis occidentalis* trees varies to favour safety or efficiency depending on site conditions

**DOI:** 10.1007/s00468-025-02603-3

**Published:** 2025-02-11

**Authors:** Kaisa Rissanen, Valentina Vitali, Daniel Kneeshaw, Alain Paquette

**Affiliations:** 1 Département Des Sciences Biologiques, Centre for Forest Research, Université du Québec À Montréal, C.P. 8888, Succursale Centre-Ville, Montréal, QC H3C 3P8 Canada; 2 Institute of Terrestrial Ecosystems, ETH Zurich, Universitätsstrasse 16, 8092 Zurich, Switzerland; 3 Swiss Federal Institute for Forest, Snow and Landscape Research WSL, Zürcherstrasse 111, CH-8903 Birmensdorf, Switzerland; 4 Present Address: Institute for Atmospheric and Earth System Research, Forest Sciences, Faculty of Agriculture and Forestry, University of Helsinki, Latokartanonkaari 7, Helsinki, Finland

**Keywords:** Wood anatomical traits, Vessel traits, Phenotypic plasticity, Urban forest, Urban tree, Xylem vulnerability

## Abstract

**Key message:**

Urban trees can acclimate to their growth environment through changes in vessel anatomy. Vessel lumen area and vessel frequency following a gradient from park trees to inner-city street trees.

**Abstract:**

Urban trees stand in potentially stressful growth environments occurring along gradients of urban heat and impermeable surface cover and, to survive, can adjust their function and structure. The consequent tree-to-tree variations in hydraulic xylem traits can shed light on tree hydraulics and capacity to acclimate to diverse conditions, as well as identify limitations to tree growth and survival. Using microscopic analysis of increment cores, we compared early wood vessel traits of the ring-porous angiosperm *Celtis occidentalis* in three urban site types: central streets, residential streets and parks, within the city of Montreal. We explored differences in vessel traits (mean vessel lumen area, vessel frequency, vessel grouping index and derived variables) between site types, vessel trait intercorrelations and correlations with monthly temperature, precipitation and heat-moisture index over 10 years. The vessel traits significantly differed between site types. Park trees had the largest and central street trees had the smallest vessel lumen area and theoretical hydraulic conductivity; traits supporting efficient water transport. Central street trees had the largest vessel frequency and smallest theoretical vulnerability to cavitation; traits connected to hydraulic safety. Residential street tree traits were in between. Among central and residential street trees, water transport efficiency traits correlated positively with cool springs or arid summers, whereas among park trees, mainly vessel frequency and grouping index responded to climate variations. These results highlight the capacity of *C. occidentalis* to acclimate to urban environments and the potential of anatomical traits for quantifying the effects of urban environments on tree functioning.

**Graphical Abstract:**

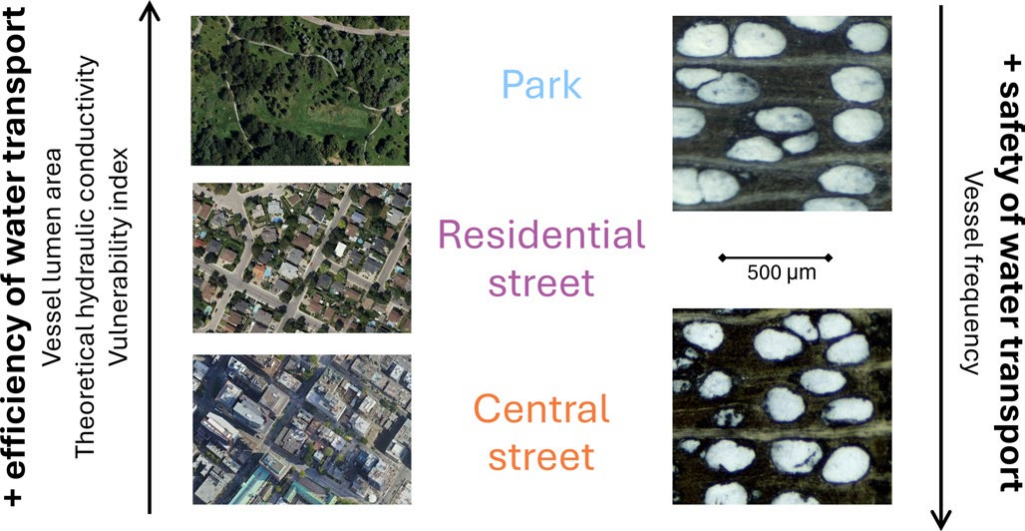

**Supplementary Information:**

The online version contains supplementary material available at 10.1007/s00468-025-02603-3.

## Introduction

The importance of urban trees for mitigating the effects of urbanisation and climate change, through cooling air by shading and transpiration, carbon sequestration and stormwater retention, is increasingly recognised (Roy et al. 2012; Livesley et al. 2016; Locosselli and Buckeridge 2023). Yet, the negative effects that trees are expected to mitigate can also limit the optimal functioning and growth of the trees themselves—and thus their capacity to provide ecosystem services. For example, the urban heat island effect (UHI) and the large extents of sealed, impermeable surfaces may increase aridity both in the atmosphere and in soils, exposing trees to drought stress (Lüttge and Buckeridge 2020). However, urban environments are complex landscape mosaics with large variations in temperature and water availability (Touchaei and Wang 2015; Gillefalk et al. 2021), yielding a variety of contrasting growth conditions (Miyahara et al. 2022). Factors such as the use of de-icing salts and exposure to mechanical damage also vary within the urban landscape and impact tree development (Vitali et al. 2019). Urban trees can acclimate to these stresses through plastic changes in their forms and functions. Along with adjustments in leaf traits and physiology (Li et al. 2020; Jang and Leung 2022) changes can occur in wood anatomy, including the size, distribution and connectivity of water conduits (Fonti et al. 2010; Gianoli and Valladares 2012). These acclimations may play an important role in how trees survive and function in different urban environments.

In natural environments, within-species variations in wood anatomy often appear over gradients of precipitation and temperature (Hacke et al. 2017). In arid versus mesic sites, the lumen areas (LA, μm^2^) of water-conducting vessels of angiosperms are smaller (Gea-Izquierdo et al. 2012; Rita et al. 2015; Castagneri et al. 2020). Frequency of vessels (VFreq, as vessels per mm^2^) is often higher in more arid sites (Gea-Izquierdo et al. 2012; Granda et al. 2018; García-Cervigón et al. 2018; Castagneri et al. 2020), with some exceptions either due to species or specific conditions of the sites (Rita et al. 2015; Tng et al. 2018). Common garden experiments have also shown differences in vessel traits between trees of the same populations planted in different climate conditions, suggesting that the variation between sites found in nature does not result solely from population-level adaptations (Schreiber et al. 2015; Lemaire et al. 2021).

Variations in vessel traits also occur over time, i.e. between growth rings of a tree, with dry years associated with smaller LA and higher VFreq (Matisons et al. 2012; Gea-Izquierdo et al. 2012; Castagneri et al. 2017, 2020; García-Cervigón et al. 2018). Furthermore, the sensitivity of vessel traits to temporal changes in environmental conditions varies between sites with differing limiting factors for vessel formation (Rita et al. 2015; Granda et al. 2018; Castagneri et al. 2020). For example, short-term variations in precipitation and drought have been reported to be more closely connected to VFreq and LA in arid compared to mesic sites (Gea-Izquierdo et al. 2012; Rita et al. 2015; Castagneri et al. 2020).

Vessel traits such as LA and VFreq, along with the characteristics of the inter-vessel pits, alter the efficiency and safety of water transport (hydraulic safety) in tree stems. The capacity of a vessel to transport water, i.e. its hydraulic conductivity, is proportional to the fourth power of its radius according to the Hagen-Poiseuille equation (Tyree et al. 1994). Therefore, for the same total lumen area, xylem with few large vessels is more efficient in transporting water than xylem with many small vessels (Tyree et al. 1994; Hacke and Sperry 2001). Simultaneously, large vessels are considered more vulnerable to cavitation and subsequent xylem dysfunction due to freezing and potentially also to drought (Hacke and Sperry 2001; Lobo et al. 2018; Lemaire et al. 2021). Still, the mechanisms linking LA to vulnerability to drought-induced cavitation are not clear (Hacke et al. 2017, Lens et al. 2022, but see Isasa et al. 2023, Olson et al. 2023). Water transport efficiency and hydraulic safety are also affected by covariation among vessel traits, as well as covariation between vessel traits and other anatomical or physiological variables (Tyree et al. 1994; Hacke and Sperry 2001; Magnani et al. 2002; Abrantes et al. 2013; Levionnois et al. 2021).

As in natural environments, wood anatomy may vary with the aridity or stress gradients in urban landscapes, allowing urban trees to acclimate through alterations in their water transport efficiency or hydraulic safety. The few studies examining wood anatomy in urban environments report trends of decreasing vessel lumen diameters (LD, μm) and hydraulic conductivity over gradients from non-urban to urban trees (de Vasconcellos and Callado 2020; da Silva et al. 2021; Gao et al. 2023), larger LD and higher VFreq among street trees in comparison to park trees (Li et al. 2020), but no relationship between LD and the degree of impermeability of the tree surroundings (Savi et al. 2015). Understanding variations in vessel traits across heterogeneous urban landscapes would be useful to describe acclimations, limitations and stresses that trees experience, and to further inform about the capacity of trees to survive, function and provide ecosystem services in varying urban but also in natural environments.

In this study, we explored the potential of urban trees to acclimate to their growth environment through plastic changes in vessel anatomy. We analysed vessel traits of a ring-porous angiosperm *Celtis occidentalis* (L.) (common hackberry) in Montreal, Canada, in three urban site types: (1) parks in residential areas, (2) lawns by residential streets, (3) inner-city street pits. From the parks to street pits, trees are more exposed to stress factors of the urban environment, including higher temperatures, soil surface impermeability and potential for mechanical damage. We hypothesised that vessel traits and their responses to year-to-year variations in temperature and precipitation differ between the three site types, potentially following a gradient from least to most exposure to urban stress factors. In addition, we explored covariations among vessel traits between and within trees.

## Methods

### Study location and study trees

The study took place in Montreal, Canada, a 1 760 000 -inhabitant city, with an annual precipitation of 1000 mm (approximately 430 mm from May to September) and an annual mean temperature of 6.8 °C. The coldest month, January, has a mean temperature of − 9.7 °C and the warmest month, July, has a mean temperature of 21.2 °C (Pierre-Elliot Trudeau airport averages over 1981–2010, https://climate.weather.gc.ca/).

**Fig. 1:**
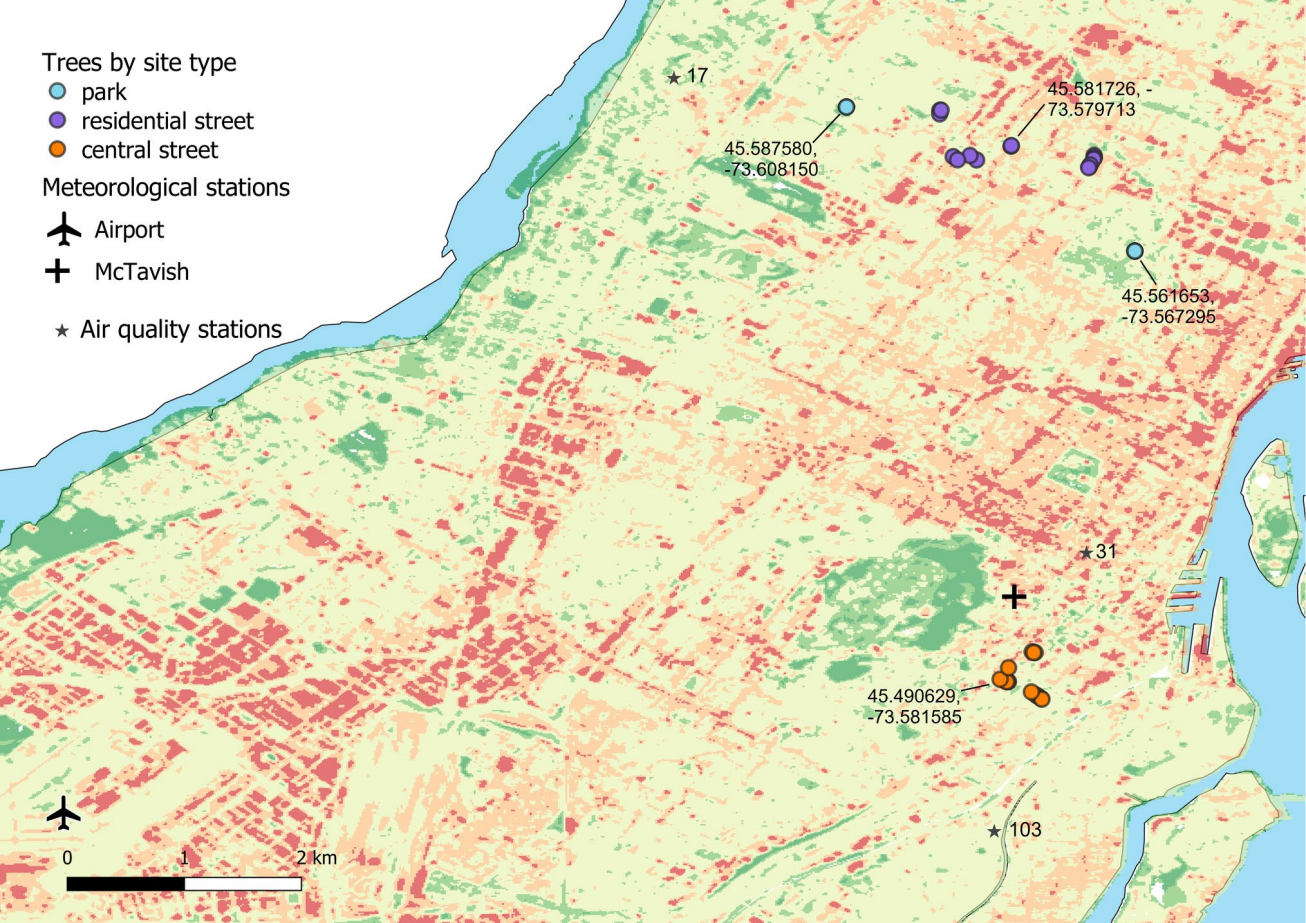
The study tree locations by site type (coordinates given for the sampling sites, EPSG: 4326—WSG 84), and locations of meteorological and air quality stations, overlayed on a map of urban heat on the island of Montreal based on 2016 high-resolution aerial image data (data source: https://donnees.montreal.ca/dataset/ilots-de-chaleur). Areas classified from dark green (cool), through pale yellow (temperature close to mean temperature) to dark red (urban heat island)

We used core samples of *Celtis occidentalis* (L.) that were acquired in 2018. *C. occidentalis* is a species with a ring-porous xylem (Wheeler et al. 1989), indigenous to the area and frequently used both as a street and park tree in the city of Montreal (3% of trees in the public tree inventory). The sampled trees were public city trees on the streets of the city centre (Ville-Marie borough), on the streets of residential areas (Saint-Léonard and Rosemont—Petite-Patrie boroughs) and in two large parks of the same residential areas (Fig. 1). The sites differed clearly in the extent of impervious surfaces around the study trees. The trees in the city centre (from here on *central street trees*) grew in pits in the sidewalk, surrounded mainly by impervious surfaces. The trees on residential streets (from here on *residential street trees*) grew on the front lawns of residential houses, but adjacent to the paved street. Park trees grew on lawns within the parks. Air quality stations nearest to the study trees (Fig. 1) revealed only small differences in the air pollution between the residential (parks and residential streets) and central areas (central streets) (Fig. S1).

For each site type (park, residential street or central street) 15 trees were selected for coring in 2018, a maximum of six trees on the same street to avoid small-scale location effects. Eleven trees were selected in de Maisonneuve Park and three in Pie IX Park. The trees were mature and established with a mean [min–max] diameter at a breast height (DBH) of 40 [32─60] cm in parks, 49 [35─66] cm on residential streets and 40 [33─50] cm on urban streets. The trees were cored at breast height from two perpendicular sides of the stem (cores A and B), avoiding scars or malformations, with a 5-mm increment borer (Haglöf Sweden AB, Langsle, Sweden). Increment cores were processed following standard dendrochronological procedures (Cook and Kairiukstis 1990) and the 20-year tree-ring width chronologies were cross-dated. For inspecting the common signal strength, we used expressed population signal (EPS) and mean inter-series correlations (r-bar and interrbar) (Table S1).

### Analysing the anatomical traits

For anatomy analysis, we used the method described by Gärtner and Nievergelt (2010) to scan the core surface. The method has been validated against thin section preparation and figure capture by light transmission microscope for species with large early-wood vessels (similar to *C. occidentalis*) by Fonti et al. (2009). First, we cleaned the core surface with a brush, stained it with a black marker and then filled the vessels with a white chalk stick. Second, we photographed the core surface using an 18MP digital camera (MU1803-HS-CK, Amscope, United Scope LLC., CA, USA) mounted on a stereo microscope (SZ-6145TR, Olympus, Tokyo, Japan) (Fig. S2). We used 45 × magnification, yielding a resolution of 1.812 px µm^−2^. Third, we used Image J software (Schneider et al. 2012) for image analysis of the 10 most recent rings per core. We focused on the areas of interest (aoi) and further analysis of the early wood because of the greater importance of early-wood vessels for water transport in ring-porous species (Fonti et al. 2010). Within the aoi, we measured the number of vessels, the vessel lumen area of each vessel, the number of vessel clusters of two or more vessels and the number of independent vessels. We excluded vessels smaller than 5000 µm^2^ (4% of all measured vessels) because they can be confused with latewood vessels or other wood structures given our image resolution (Fonti and García-González 2004; Castagneri et al. 2017).

From the measured vessels, we calculated the following vessel traits (Table 1) first per tree, core and tree ring (A and B cores separate), and then per tree and tree ring (combining the A and B cores):

i. The mean vessel lumen area (LA, µm^2^) which strongly affects xylem water transport capacity and may vary with environmental conditions (Gea-Izquierdo et al. 2012; Castagneri et al. 2020).
ii. The vessel frequency (VFreq, n of vessels in aoi, n mm^−2^) which also affects water transport capacity and its redundancy (Fonti and García-González 2004; Gea-Izquierdo et al. 2012; Abrantes et al. 2013). Additionally, vessel fraction (VFrac, total vessel lumen area/aoi, µm^2^ µm^−2^), which describes the ring surface covered by vessels.
iii. The hydraulically weighted vessel diameter (HWD, µm; Sperry and Saliendra 1994) and theoretical hydraulic conductivity of xylem (assuming round vessels) based on the Poiseuille’s law (THC, Kg m^−1^ MPa^−1^ s^−1^; Tyree and Zimmermann 2002), as proxies for water transport efficiency. For water transport safety, we calculated the vulnerability index (VI) for the theoretical vulnerability of vessels to cavitation (vessel diameter/ Vfreq; Carlquist 1977).
iv. The vessel grouping index (GI n of vessels / n of vessel groups, including solitary vessels; Scholz et al. 2013) for which larger values indicate more vessels in groups, and the solitary vessel index (SVI, n of solitary vessels / n of vessels; von Arx et al. 2013), for which larger values indicate a larger proportion of solitary vessels. Vessel connectivity or grouping may also affect the vulnerability of the xylem to cavitation through redundancy in water transport and through ease of air penetration to neighbouring vessels.

**Table 1:** ANOVA F-tests for LME testing the site type effects on log or square root transformed vessel traits, and the non-transformed mean values and standard deviation (SD) of the vessel traits per location, calculated from all measurements

Vessel trait	Formula (unit)	ANOVA F-test for site type effect		Mean per site type (SD)		
		*F*	*p* value	Park	Residential st	Central st
Mean vessel lumen area (LA)	(µm^2^)	14.8	< 0.001	24,920^a^ (7008)	21,980^b^ (4991)	18,530^c^ (5416)
Vessel fraction (VFrac)	total vessel lumen area/aoi (µm^2^ µm^−2^)	1.20	0.31	0.270 (0.061)	0.271 (0.043)	0.253 (0.049)
Vessel frequency (VFreq)	*n* of vessels/aoi (n mm^−2^)	10.4	< 0.001	11.3^a^ (2.52)	12.7^b^ (2.39)	14.3^b^ (3.29)
Hydraulically weighted vessel diameter (HWD)	∑D^5^ /∑D^4^ (µm)	10.7	< 0.001	201^a^ (38.8)	185^b^ (22.3)	172^b^ (26.9)
Theoretical hydraulic conductivity of xylem (THC)	π∑D^4^ρ/128ƞaoi (Kg m^−1^ MPa^−1^ s^−1^)	8.9	< 0.001	330^a^ (192)	274^ab^ (86.5)	224^b^ (91.0)
Vulnerability index (VI)	mean D/VFreq	13.5	< 0.001	16.9^a^ (6.74)	13.9^b^ (4.85)	11.5^b^ (4.78)
Vessel grouping index (GI)	*n* of vessels/*n* of vessel groups (incl. solitary)	1.91	0.16	1.25 (0.12)	1.25 (0.12)	1.21 (0.18)
Solitary vessel index (SVI)	*n* of solitary vessels/*n* of vessels	0.01	0.99	0.595 (0.121)	0.598 (0.127)	0.594 (0.137)

LME function form in R (lme4 package): lme(var~site type, random=~1|tree_id/core)

D=single vessel diameter (calculated from lumen area assuming a round vessel)

Ƞ=the viscosity of water at 20°C (1.002 × 10‐9 MPa s)

ρ=the density of water at 20°C (998.2 kg m−3)

aoi=area of interest

At each site type, n_trees_ = 14─15, and up to ten latest growth rings per tree and per core were considered. Differing letters after the mean value indicate significant (p < 0.05) differences between the site types according to post-hoc tests with Bonferroni correction. For ANOVA tests, numerator DF = 612–620 and denominator DF = 40

We inspected the common signal strength of the 10-year LA, VFreq and GI chronologies similarly to the tree-ring width chronologies (Table S1).

### Meteorological data

We accessed daily meteorological data (mean, maximum and minimum temperatures, precipitation sum, https://climate.weather.gc.ca/) from the Montreal-Pierre Elliott Trudeau International Airport meteorological station to correspond to the park and residential street trees, and from the McTavish meteorological station in the city centre to correspond to the central street trees (Fig. 1, see also Fig. S3).

### Data analysis

#### Site-type differences in vessel traits

We used linear mixed-effect models (LMEs, lme function of R-package nlme v 3.1.160; Pinheiro et al. 2020) to analyse the effect of site type (i.e., park, residential street or central street) on each vessel trait (Table 1), using the vessel trait values per tree, tree core (A or B) and ring as the response variables. We used site type as a fixed effect, and tree ID and the tree core nested in tree ID as random effects. We assessed the normality of model residuals with QQ-plot visual inspection and applied a log or square-root transformation to the original data where it improved residual normality. In case of a significant site type effect, we conducted post-hoc tests with Bonferroni correction to examine the specific differences between site types (emmeans function of R-package emmeans v 1.8.5; Russel 2022). We selected Bonferroni correction to accommodate the differing variance between site types (assessed by Levene test, leveneTest function of R-package car v 3.1.1; Fox and Weisberg 2019).

#### Correlations among vessel traits and between vessel traits and meteorological variables

For all correlation analyses, we used the vessel trait values per tree and tree ring (A and B cores combined). To explore the relationships between vessel traits, we first calculated Pearson’s correlations between the measured vessel traits: LA, VFreq, VFrac, and the grouping indices: GI, SVI, using the tree-wise mean values for between-tree correlations. Second, we calculated repeated measures correlations (rmcorr, r-package rmcorr vs 0.5.4; Bakdash and Marusich 2017) with tree ID as subject (i.e. random effect) to explore within-tree correlations. We calculated both correlations across all trees, and separately for the three site types.

To explore how vessel traits respond to variations in environmental conditions, we tested for temporal associations between the vessel traits and meteorological variables. We did this by first calculating the precipitation sum, mean temperature and heat moisture index (HMI, as in Wang et al. 2006 and Chavardès et al. 2013) from the previous year May to December and the current year January to July in 30-day windows that moved in 15-day steps, meaning that each window covered either one month or the end and beginning of two consecutive months. We then detrended both the climate data and the vessel trait data by taking the first differences. Finally, we calculated rmcorr between the detrended vessel traits and the detrended meteorological variables (precipitation, temperature, HMI) with the tree ID as the subject, separately for each site type. We also calculated rmcorr using non-detrended and linearly detrended vessel trait values and meteorological variables, but these did not provide additional information to the first difference detrended data and thus we do not discuss them further.

#### Tree height impact on vessel traits

Vessel lumen area increases with distance to the treetop to counteract resistance in the hydraulic pathway (Carrer et al. 2015), which affects the interpretation of mean vessel lumen areas between trees of different heights. As tree heights were not measured in 2018, we returned to the sampling locations in 2023 and measured the heights of the sampled trees in residential and central streets. In parks, we measured a group of 10 representative trees (similar DBH-range to the sample trees), because the original trees could not be identified. We then tested the difference in the 2023 tree heights between site types. The mean tree heights only marginally differed between site types, with trees being slightly taller in parks compared to central streets (ANOVA F_2, 35_ = 2.95, p = 0.07, Fig. S4).

To further explore the potential impact of tree size on the observed vessel traits, we calculated Pearson’s correlations of the tree-wise mean of measured vessel traits with tree DBH measured in 2018, and tree height measured in 2023 (for central and residential trees only), both over all trees and by site type. Finally, using residential and central trees, we combined site type and tree height in 2023 as fixed effects and tree ID and the tree ring-core nested in tree ID as random effects in LMEs explaining each vessel trait.

## Results

### Vessel trait differences between site types

**Fig. 2:**
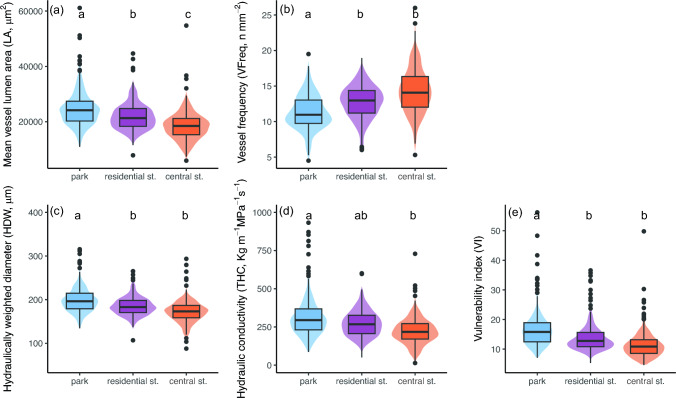
Site type differences in vessel traits for which significant differences from Table 1 were observed for *C. occidentalis*. For each site type, n_trees_ = 14─15, the ten latest growth rings per tree and per core were considered. The filled area describes data distribution in each category, the box upper and lower limits give the lower and upper quartiles of data distribution, and the mid-lines give median. The whiskers give maximum and minimum values, excluding outliers shown as black dots. Differing letters indicate significant (*p* < 0.05) differences in group means according to post-hoc test with Bonferroni correction, see Table 1

The vessel traits varied between the site types, with mainly park trees differing significantly from the residential and central street trees (Fig. 2, Table 1). Mean vessel lumen area (LA) was largest in park trees and smallest in central street trees, with residential street trees in the middle (Fig. 2a, Table 1). Both hydraulically weighted vessel diameter (HWD) and theoretical hydraulic conductivity of xylem (THC), were also largest in park trees, but without significant differences between residential and central street trees (Fig. 2c, d, Table 1). On the contrary, vessel frequency (VFreq) was smallest in park trees and larger in residential and central street trees (Fig. 2b, Table 1). Vulnerability index (VI) was consequently highest in park trees and not significantly different between residential and central street trees (Fig. 2e, Table 1). For vessel fraction (VFrac), vessel grouping index (GI) and solitary vessel index (SVI), the site type effect was not significant (Table 1).

### Tree height impact on vessel traits

We found no significant correlations between the tree-wise mean vessel traits and tree DBH, but tree height in 2023 correlated negatively with VFreq over residential and central street trees (Table S3). However, LME combining height and site type effects showed no significant marginal effect of height on vessel traits among residential and central street trees. The marginal effect of site type was significant for LA and THC (Table S4). Thus, although the small, marginally significant differences in tree heights between site types may have impacted vessel traits due to the tapering of LA with hydraulic path length, the site types affected the vessel traits independently.

### Correlations among vessel traits and between vessel traits and tree size

**Fig. 3:**
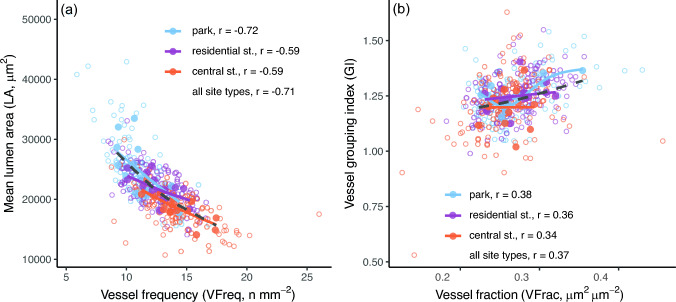
Relationship between **a** the mean vessel lumen area (LA) and vessel frequency (VFreq), and between **b** vessel grouping index (GI) and vessel fraction (VFrac) in *C. occidentalis* trees over three locations in Montreal. Open symbols show all ten rings per tree, filled symbols show the tree-wise mean values. Lines depict the relationship trend per site type (different colours) and overall site types (black dashed line) for tree-wise mean values. Correlation coefficients (r) for Pearson’s correlations between the tree-wise mean values are given (see also Table 2). Repeated measures correlation coefficients for within-tree correlations are in Table S2

Tree-wise mean values of LA and VFreq had a clear negative correlation both over all trees and within each site type (Fig. 3a, Table 2), following the trend observed between the site types (Fig. 2a, b, Table 1). Both LA and VFreq also inherently correlated positively with VFrac (Table 2), while the vessel grouping indices GI and SVI correlated negatively (Table 2). GI also correlated positively with VFrac over all trees (Fig. 3b, Table 2), with LA in residential and central street trees (Table 2) and with VFreq in park trees (Table 2). SVI correlated negatively with VFrac and VFreq (Table 2). The repeated measures correlations using all rings per tree, concentrating on the within-tree correlations between traits, showed similar relationships between the traits as Pearson’s correlations with the tree-wise mean values (Table S2, Fig. 3). As an exception, park tree GI was positively associated with VFreq but not with VFrac in within-tree correlations (Table S2).

**Table 2:** Pearson’s correlations for relationships between the tree-wise means of vessel traits over all trees and by site type

	LA	VFrac	VFreq	GI
All trees				
VFrac	0.45			
VFreq	− 0.71	0.25		
GI	0.17	0.37	(0.02)	
SVI	(0.07)	− 0.23	− 0.22	− 0.82
Park				
VFrac	0.50			
VFreq	− 0.72	(0.17)		
GI	(0.01)	0.38	0.22	
SVI	(0.10)	− 0.30	− 0.27	− 0.92
Residential street				
VFrac	0.44			
VFreq	− 0.59	0.45		
GI	0.16	0.36	(0.13)	
SVI	(− 0.01)	− 0.31	− 0.25	− 0.87
Central street				
VFrac	0.45			
VFreq	− 0.59	0.43		
GI	0.23	0.34	(0.05)	
SVI	(0.11)	(− 0.11)	− 0.21	− 0.79

At each site type, n_trees_ = 14─15, and up to ten latest growth rings per tree and per core were used for calculating tree-wise mean. Measured vessel traits: LA = mean vessel lumen area, VFrac = vessel fraction, VFreq =  vessel frequency, vessel grouping indices: GI = vessel grouping index,  SVI = solitary vessel index. All correlations are significant at p < 0.05 unless the correlation coefficient is in parenthesis. For repeated measures correlations with within-tree correlations, see Table S2

### Vessel trait responses to variations in precipitation, temperature and HMI

**Fig. 4:**
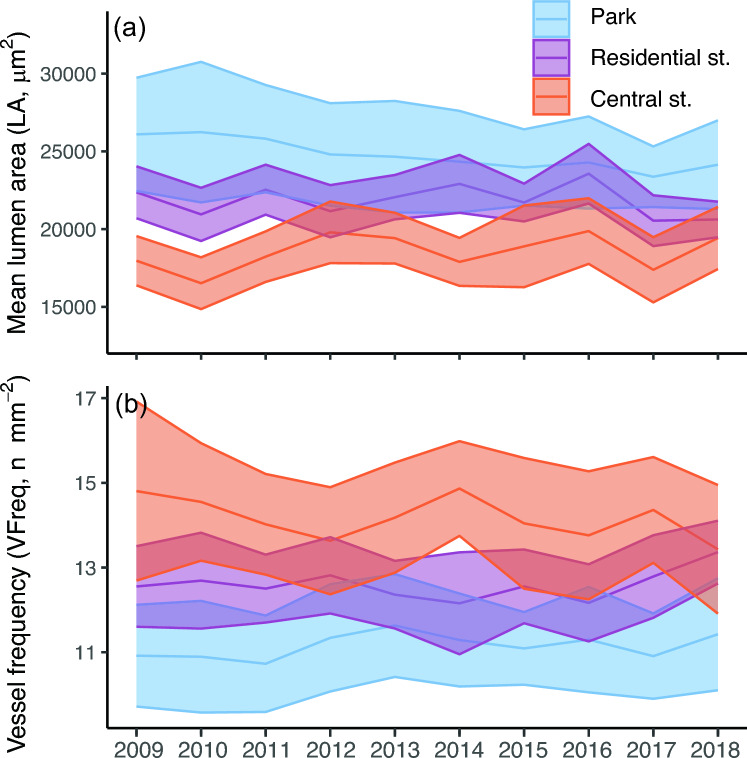
Non-detrended year-to-year variation in **a** the mean vessel lumen area (LA) and **b** vessel frequency (VFreq) of *C. occidentalis* in Montreal, the middle line per location depicting the mean per year and the filled area the confidence interval (at 95%), each colour representing a different site type

The vessel traits varied in time through the 10 years and the year-to-year variation in both LA and VFreq seemed most pronounced among central street trees (Fig. 4). Among park trees, the amplitude of year-to-year variation in LA was potentially dampened by the large differences between individual trees as shown by the wider confidence interval (Fig. 4a). The population signals for the vessel trait time series were relatively low particularly among park trees (Table S1), so we consider the climate-correlations presented below to be tentative.

**Fig. 5:**
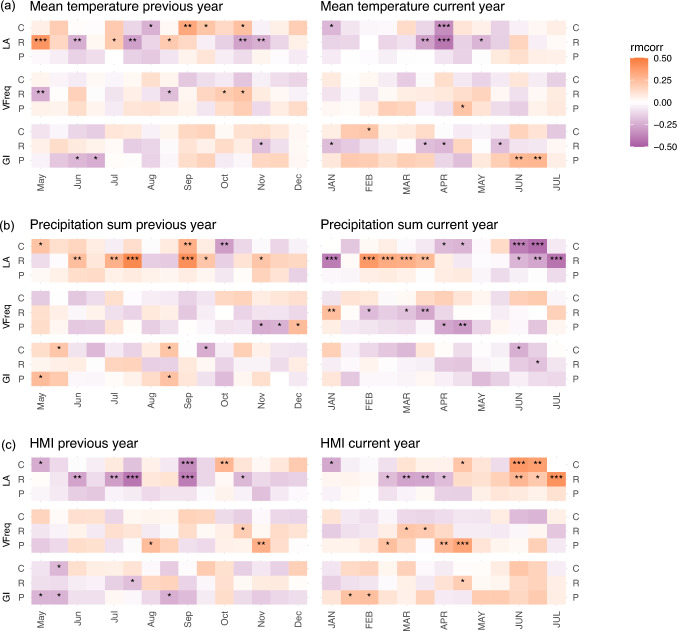
Repeated measures correlation coefficients (rmcorr) between selected detrended vessel traits (LA = mean vessel lumen area, VFreq = vessel frequency, GI = vessel grouping index) and detrended mean **a** temperature, **b** precipitation or **c** heat-moisture index (HMI) of 30-day periods moved in 15-day steps from May to December of previous year (lower case letters) and from current year (year corresponding to the growth ring formation) January to July (upper case letters). Correlation significance: *p < 0.05, **p < 0.01, ***p < 0.001; Site types: *P* park, *R* residential street, *C* central street. For figures with all vessel traits, see Fig. S5─S7

In both residential and central street trees, LA correlated negatively with current year spring (late March–April) temperature and summer (June–July) precipitation (Fig. 5a, b). In addition, LA of residential street trees correlated positively with the current year’s late winter and early spring (February–early April) precipitation, while LA of central street trees correlated negatively with spring (April–early May) precipitation (Fig. 5b). Consequently, LA of residential street trees correlated negatively with current year spring (March–April) HMI, and LA in both residential and central street trees correlated positively with summer (June–July) HMI (Fig. 5c). The correlations of LA with previous year conditions varied, showing mainly negative associations with HMI and temperature, and positive associations with precipitation of the previous year summer (June–early August) (Fig. 5). THC, HWD, VI and VFrac showed generally similar correlation patterns with LA among central and residential street trees (Fig. S5─S7).

In park trees, the correlations of LA, THC, HWD, VI and VFrac with the current year temperature, precipitation and HMI were non-existent or weaker than among the central and residential street trees (Fig. 5, Fig. S5-S7). However, park tree VI correlated positively with precipitation and negatively with HMI and park tree VFrac correlated negatively with precipitation and positively with HMI of the current year spring (April and May) (Fig. S6-S7).

In comparison to LA, VFreq was less connected to the previous and current year temperature and precipitation patterns, in particular among central street trees (Fig. 5a, b). However, VFreq correlated negatively with precipitation and positively with HMI of current-year spring (March–early April for residential, April–early May for park trees) among both residential street trees and park trees (Fig. 5b, c). Previous autumn (late October–November) temperatures and HMI had positive correlations with park and residential street tree VFreq (Fig. 5a, c).

GI of central street trees correlated only weakly with temperature and precipitation patterns of the current year, but it did correlate positively with precipitation of the current year June (Fig. 5a, b). Among residential street trees, GI correlated negatively with current year spring (late March–April) temperature but positively with later spring (late April–early May) HMI (Fig. 5c). In contrast to street trees, among park trees GI correlated positively with current year summer (June–early July) temperature (Fig. 5a). The correlation patterns of SVI with temperature, precipitation or HMI generally largely followed the patterns observed for GI but inversely (Fig. S5─S7).

## Discussion

In this study, *Celtis occidentalis* vessel traits significantly differed between urban site types (park, residential street and central street), highlighting the capacity of urban trees to acclimate to the distinct limitations of growing environments within a city. The studied site types also showed differing sensitivities of vessel traits to temperature and precipitation patterns. In street site types, mainly mean vessel lumen area (LA) responded to variations in climate, whereas in parks, vessel frequency (VFreq) and grouping index (GI) were most sensitive to climate variations.

### Vessel traits differ between urban site types

We found that from park trees to residential street trees and central street trees, there was a decrease in traits promoting efficient water transport (mean vessel lumen area (LA), hydraulically weighted diameter (HWD) and theoretical hydraulic conductivity of xylem (THC)), and an increase in vessel frequency (VFreq) (Table 3). These results suggest that plastic adjustments in wood anatomy play a role in the acclimation of planted trees to their urban growth environments, similarly to the plasticity in leaf traits (Esperon-Rodriguez et al. 2020; Ibsen et al. 2023). Similar results along urban gradients have been shown in LA, THC and VFreq for (i) *Guarea guidonia* from a forest to a peri-urban and an urban park (da Silva et al. 2021), (ii) *Ceiba speciosa* from a forest to trees along a large avenue (de Vasconcellos and Callado 2020) and (iii) *Quercus mongolica* and *Fraxinus mandshurica* from rural to urban forest (Gao et al. 2023). Li et al. (2020) showed that for some vessel traits, in particular VFreq, the differences between park and street trees were species-dependent, suggesting that the vessel-based acclimation strategies between species may vary and calling for further research to discover potential patterns across species.

**Table 3:** Summary of the change in vessel traits from park trees to residential street trees and central street trees

	Vessel trait	Direction of increase
Park		
	LA	↑
	THC	↑
Residential street	HWD	↑
	VI	↑
	VFrac	-
Central street	GI	-
	SVI	-
	VFreq	↓

− indicates no significant change. LA = mean vessel lumen area, THC = heoretical hydraulic conductivity, HWD = hydraulically weighted diameter, VI = vulnerability index, VFrac = vessel fraction, GI = vessel grouping index, SVI = solitary vessel index, VFreq = vessel frequency

The LA variation that we observed between site types could also partially result from tree height differences. However, we observed only small differences in mean tree heights between the site types (see Fig. S4) along with independent site-type effects on LA and THC even when tree height was considered (Table S4), both indicating that the site-type effects on vessel traits were not caused by tree height differences.

Our results on the change of vessel traits from park trees to central street trees can be considered analogous to the gradient from mesic to arid sites found in forest environments. In forest environments, trees on arid sites tend to have vessel anatomy which is less efficient but potentially better protected against cavitation (e.g. high VFreq, low VI), while trees on mesic sites tend to have vessel traits that support better water transport efficiency (e.g. large LA and THC) (Gea-Izquierdo et al. 2012; Rita et al. 2015; Tng et al. 2018; Granda et al. 2018; Castagneri et al. 2020). Similar trends have also been found when comparing street trees between cities of varying aridity (Zhu et al. 2023). Within a city, central streets correspond to arid sites as trees are most exposed to high temperatures and consequently higher VPD, and are surrounded by impermeable surfaces that limit their access to rainwater and expose them to stronger drought conditions (Yang and Zhang 2011; Savi et al. 2015; Rötzer et al. 2021). On the contrary, park trees can be expected to have better access to soil and rainwater and experience lower temperatures, therefore, being more similar to mesic sites (Bowler et al. 2010). Finally, residential street trees are somewhere in between, both affected by the close-by paved street and heat of the built environment and profiting from permeable surfaces.

In addition to temperature and water availability, urban environments are characterised by other factors, such as artificial lighting, exposure to salt and other pollutants, pruning intensity and the potential for mechanical damage. These factors also vary between urban site types and could cause changes or damage in tree structures, and understanding their impacts on tree anatomy and growth would help refine tree management strategies. Yet the specific effects of mechanical or pollution-related stress factors on anatomy have been studied little (but see Rajput et al. 2008; Rinne et al. 2011; Vitali et al. 2019).

### Correlations among vessel traits

In addition to individual vessel traits, their interactions also play a role in tree hydraulics and acclimation to dry conditions (Abrantes et al. 2013; García-Cervigón et al. 2018). We observed the often-reported strong negative correlation between LA and VFreq (Fonti and García-González 2004; Zanne et al. 2010; Scholz et al. 2014; Castagneri et al. 2020). The covariance of LA and VFreq has been suggested to stabilise or guarantee an adequate total conductive lumen area even when LA decreases (García-Cervigón et al. 2018), although the increase in VFreq would need to be considerable to counteract the adverse impact of reduced LA on hydraulic conductivity (Tyree et al. 1994). The correlations between LA and VFreq were consistent between site types both within and between trees, which suggests that the mechanisms driving the coordination between these traits vary little between site types. Indeed, the relationship is at least partly a simple result of a space constraint: larger vessel lumen areas reduce the number of vessels that fit per xylem surface area (i.e. vessel frequency).

Additionally, we observed that the GI correlated positively with vessel faction (VFrac), which seemed to be driven by a positive correlation between GI and LA in central and residential street trees and by a positive correlation between GI and VFreq in park trees. Similar to our findings with park trees, Levionnois et al. (2021) observed across species an increase in GI with an increase in VFreq, but contrary to our findings with street trees, a decrease in GI with an increase in LA. There are no clear mechanistic explanations for the positive GI-VFrac correlation. On one hand, a larger fraction of vessels, be it due to higher LA or VFreq, reduce the space between vessels, thus potentially promoting more vessel grouping. On the other hand, increased vessel grouping is hypothesised to associate with higher embolism resistance by offering alternative pathways for water and mitigating the loss of water transport capacity from cavitation (Hacke and Sperry 2001; Tyree and Zimmermann 2002; Levionnois et al. 2021) or on the contrary to allow easier spread of embolism to adjacent vessels and reduce xylem embolism resistance (Avila et al. 2023). If vessel grouping increases hydraulic safety, the increase of GI with an increase in LA in our central and residential street trees could be a mechanism to counteract the higher risk for cavitation.

### Vessel trait responses to temporal variation in precipitation, temperature and HMI

*C. occidentalis* in our urban environments had generally relatively low common signals in tree-ring and vessel trait chronologies, likely because of the short chronologies (20 years for tree-ring width and 10 years for vessel traits) and the local-scale urban impacts such as construction that can add noise to growth and vessel formation. Yet, we observed tentative correlations between vessel traits and year-to-year variations in meteorological conditions, and differences in the correlations between site types. Most notably, the vessel traits describing water transport efficiency (LA, THC, HWD, VFrac) responded to changes in meteorological conditions among central and residential street trees, but not among park trees. Site-type differences in vessel trait responses to meteorological conditions have been observed before, for example, Rita et al. (2015) and Castagneri et al. (2020) reported weaker effects of precipitation on vessel lumen areas in sites with higher humidity, corresponding to our park sites.

Among central and residential street trees, the vessel traits describing water transport efficiency tended to respond negatively to spring temperatures and positively to summer aridity. Similar to our findings, negative responses of vessel lumen areas of ring-porous species (*Castanea saliva, Quercus petraea, Quercus rubra*) to early spring temperature and spring and summer precipitation have been reported in temperate regions (Switzerland, Fonti and García-González 2004, 2008, and Latvia, Matisons et al. 2015). Rather than with changes in water transport efficiency, park trees in our study seemed to react to spring aridity or high summer temperatures with increased VFreq or GI, so with increased redundancy of the water-conducting system. The observed year-to-year variability of GI suggests that despite appearing consistent across the three site types, the vessel grouping pattern may not be a fixed trait of the species.

As a ring-porous species, *C. occidentalis* uses the carbon reserves stored the previous year to build early wood and the first vessels (Barbaroux and Bréda 2002; Zweifel et al. 2006). We thus expected the conditions of the previous growing season to also significantly affect the vessel traits. In both street site types, we observed increased LA in association with decreasing temperatures of the previous spring and increasing temperatures of the previous autumn. In addition, among residential street trees, increased LA was associated with decreased aridity in the previous year, as also reported by Fonti and García-González (2008) and Matisons et al. (2012, 2015). Thus, for street trees, in residential areas in particular, humid conditions of the previous year’s summer and warmth in the previous year’s autumn appear to predict larger efficiency in water transport in the current year.

## Conclusions

In conclusion, we found that the ring-porous *C. occidentalis* vessel traits varied along a gradient of urban site types from parks (with the least impact of urban stress factors) to residential streets and to central streets (with the most impact of urban stress factors) and that the responses of vessel traits to previous and current year meteorological conditions also tended to differ between site types. These differences are likely caused by different limiting factors of the environment, such as water (Rita et al. 2015; Castagneri et al. 2020), and by additional chemical and mechanical impacts (Rajput et al. 2008; Vitali et al. 2019). Whether these results also apply to angiosperms with diffuse-porous wood should be a subject of further research. Our results, together with an increased understanding of the variation in other physiological, anatomical and morphological adjustments of urban trees (Jang and Leung 2022), can help to explain how trees survive or even thrive in urban conditions and in corresponding natural environments. How trees experience and acclimate to different urban site types is also valuable information for the planning of tree planting and management strategies. For example, in the particular case of Montreal, we note that park trees were theoretically most vulnerable to cavitation and thus to a reduction of water transport capacity in intense or repeated dry periods. On the contrary, central street trees were theoretically the least vulnerable to cavitation but had the lowest water transport capacity and thus smallest potential for cooling by transpiration in non-drought conditions.

## Supplementary Information

Below is the link to the electronic supplementary material.

Supplementary file1 (PDF 552 KB)

## Data Availability

The data underlying this article will be shared on reasonable request to the corresponding author.
