# Supplementary material for: Vessel anatomy of urban Celtis occidentalis trees varies to favour safety or efficiency depending on site conditions
Source: Trees (Berl West). 2025 Feb 11;39(1):29. doi: 10.1007/s00468-025-02603-3 (PMC11813969; doi:10.1007/s00468-025-02603-3)
Supplement: Supplementary file 1 — Supplementary file1 (PDF 552 KB) [file 468_2025_2603_MOESM1_ESM.pdf]

Supplementary Information for

Vessel anatomy of urban *Celtis occidentalis* trees varies to favour safety or efficiency depending on site conditions

(Trees – Structure and Function)

Kaisa Rissanen[1]*, Valentina Vitali, Daniel Kneeshaw & Alain Paquette

*Corresponding author, present contact information:

Institute for Atmospheric and Earth System Research / Forest Sciences, Faculty of Agriculture and Forestry, Latokartanonkaari 7, University of Helsinki, Helsinki, Finland

kaisa.rissanen@helsinki.fi

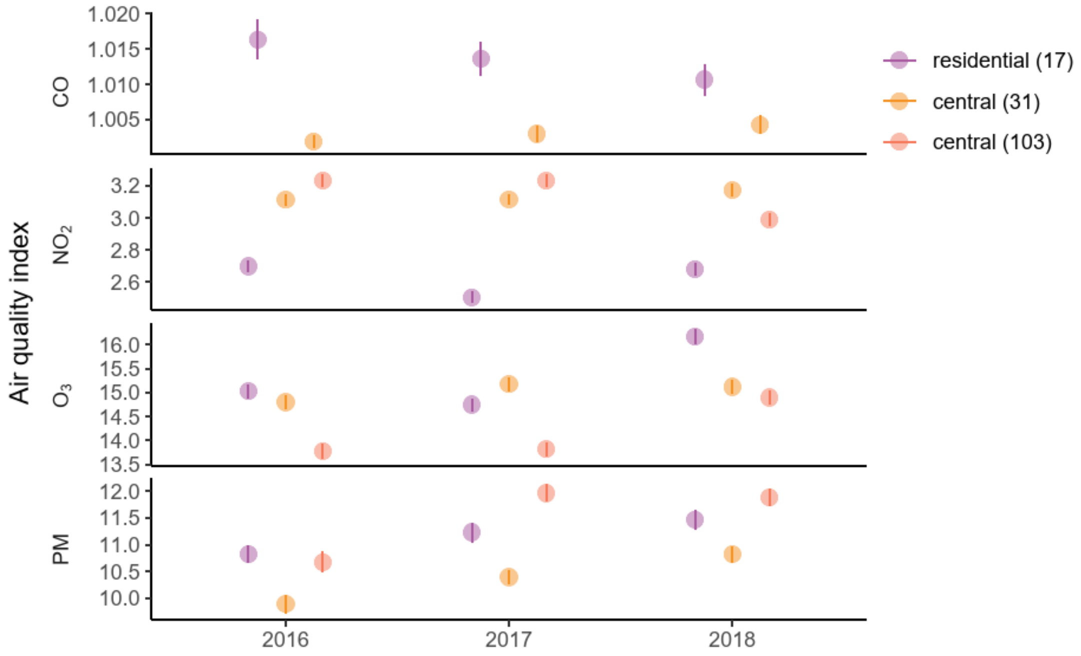

Fig. S1 Yearly mean air quality index (good: 1—25, acceptable: 26—50, bad: > 50) for carbon monoxide (CO), nitrogen dioxide (NO_2), ozone (O_3) and particulate matter (PM), means over the period 2016—2018 measured at air quality stations in the city of Montreal (RSQA). The stations shown were closest to the study trees (Fig. 1), the station in the residential area corresponds to the park and residential street trees and stations in the central areas to central street trees. The error bars give confidence intervals for yearly mean values. For more information on the air quality index, see https://donnees.montreal.ca/dataset/rsqa-iqa-historique (last accessed 31.10.2024)

Table S1 The indices of common signal quality including tree-ring width data from 1999 to 2018, and vessel lumen area (LA), vessel frequency (VFreq) and grouping index (GI) data from 2008 to 2018. GLK=mean of Gleichläufigkeit agreement test, eps=expressed population signal, rbar=mean inter-series correlation, interrbar=mean correlation between all series and mean chronology. Number of trees included in each site type=14-15.

	glk (10-yr)	eps	rbar	interrbar
**Ring width**				
Park	0.55	0.58	0.09	0.19
Residential street	0.54	0.31	0.03	0.08
Central street	0.53	0.73	0.15	0.14
**LA**				
Park		-0.47	-0.04	-0.06
Residential street		0.71	0.15	0.24
Central street		0.61	0.12	0.24
**VFreq**				
Park		0.37	0.062	-0.16
Residential street		0.13	0.01	0.05
Central street		0.11	0.01	0.10
**GI**				
Park		0.45	0.08	0.18
Residential street		0.041	0.00	0.14
Central street		-0.17	-0.01	0.18

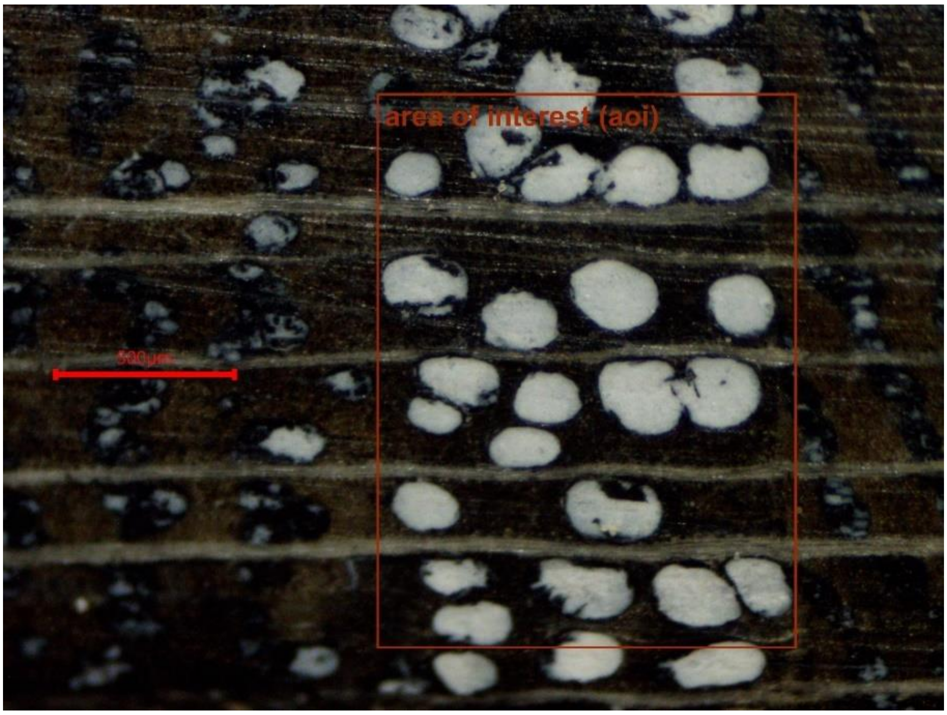

Fig. S2 An example of a photo and the area of interest used in analysing the vessel traits

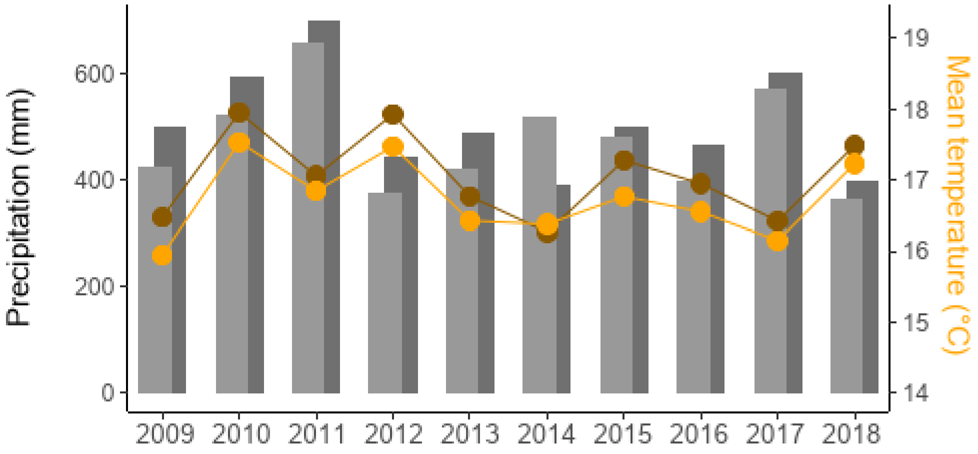

Fig. S3 Summertime (April-August) total precipitation (light grey bars for airport and dark grey bars for city centre) and mean temperature (yellow points for airport and brown points for city centre)

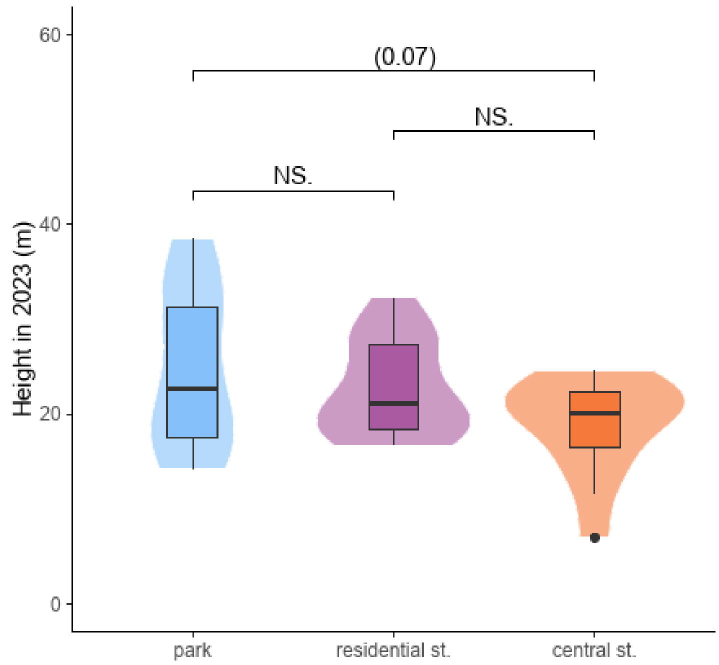

Fig. S4 Tree height differences between site types in 2023, using 10-14 trees of similar DBH range as the 2018 sample trees per site type. The height differences between park and central street trees were marginal according to ANOVA F-test (DF=35, F-value=2.95, p=0.07). p-values over brackets give the p-values for post-hoc test with Bonferroni correction (ns. = not significant)

Table S2 Repeated correlation coefficients for relationships between vessel traits for all trees, separated by site type, with tree ID as the subject (i.e. random effect). At each site type, n_{trees}=14-15, the ten latest growth rings per tree and per core were considered. Measured vessel traits: LA=vessel lumen area, VFrac=vessel fraction, VFreq=vessel frequency, vessel grouping indices: GI=vessel grouping index, SVI=solitary vessel index. All correlations as significant at p<0.05 unless the correlation coefficient is in parenthesis

	LA	VFrac	VFreq	GI
**All trees**				
VFrac	0.37			
VFreq	-0.59	0.44		
GI	(0.05)	0.31	0.12	
SVI	0.14	-0.12	-0.17	-0.81
**Park**				
VFrac	0.24			
VFreq	-0.79	0.29		
GI	-0.32	(0.01)	0.29	
SVI	0.37	(0.01)	-0.31	-0.91
**Residential street**				
VFrac	0.54			
VFreq	-0.59	0.34		
GI	0.21	0.37	(0.06)	
SVI	(-0.02)	-0.23	(-0.15)	-0.82
**Central street**				
VFrac	0.47			
VFreq	-0.46	0.54		
GI	0.31	0.40	(0.07)	
SVI	(-0.04)	(-0.14)	(-0.09)	-0.77

Table S3 Pearson's correlation coefficients between tree-wise means of measured vessel traits (LA=vessel lumen area, VFreq=vessel frequency, GI=vessel grouping index) and tree diameter at breast height (DBH) measured in 2018 or tree height measured in 2023 (only residential and central street trees), * correlation was significant at p<0.05, (*) correlation was marginally significant at p<0.1

	LA	VFreq	GI
**DBH (measured 2018)**			
All trees (n=43)	0.17	-0.17	0.01
Park trees (n=14)	0.26	-0.09	0.12
Residential street (n=15)	0.34	-0.24	-0.45 (*)
Central street (n=14)	-0.01	-0.39	0.03
**Height (measured 2023)**			
All trees (n=26)	0.38 (*)	-0.44 *	0.34 (*)
Residential street (n=14)	0.38	-0.30	0.13
Central street (n=13)	-0.10	-0.31	0.37

Table S4 ANOVA type II F-tests and p-values for LME testing tree height (measured in 2023) and site type (only residential and central street trees) effects on log or square root transformed vessel traits (see Table 1). Significant marginal effects (p<0.05) are highlighted in bold. At each site type, n_{trees}=14–15, and up to ten latest growth rings per tree and per core were considered. Numerator DF=404-407 and denominator DF=23. (LA=mean vessel lumen areas, THC=theoretical hydraulic conductivity, HWD=hydraulically weighted diameter, VI=vulnerability index, VFreq=vessel frequency, VFrac=vessel fraction, GI=vessel grouping index, SVI=solitary vessel index)

	Height (2023)		Site type (residential or central street)	
	F	P	F	P
LA	0.75	0.392	6.09	**0.022**
VFrac	0.61	0.444	3.13	0.090
VFreq	2.60	0.120	1.03	0.321
HWD	0.83	0.373	3.39	0.079
THC	0.03	0.864	6.50	**0.018**
VI	2.07	0.164	2.54	0.125
GI	0.23	0.634	1.30	0.266
SVI	0.39	0.536	0.12	0.736

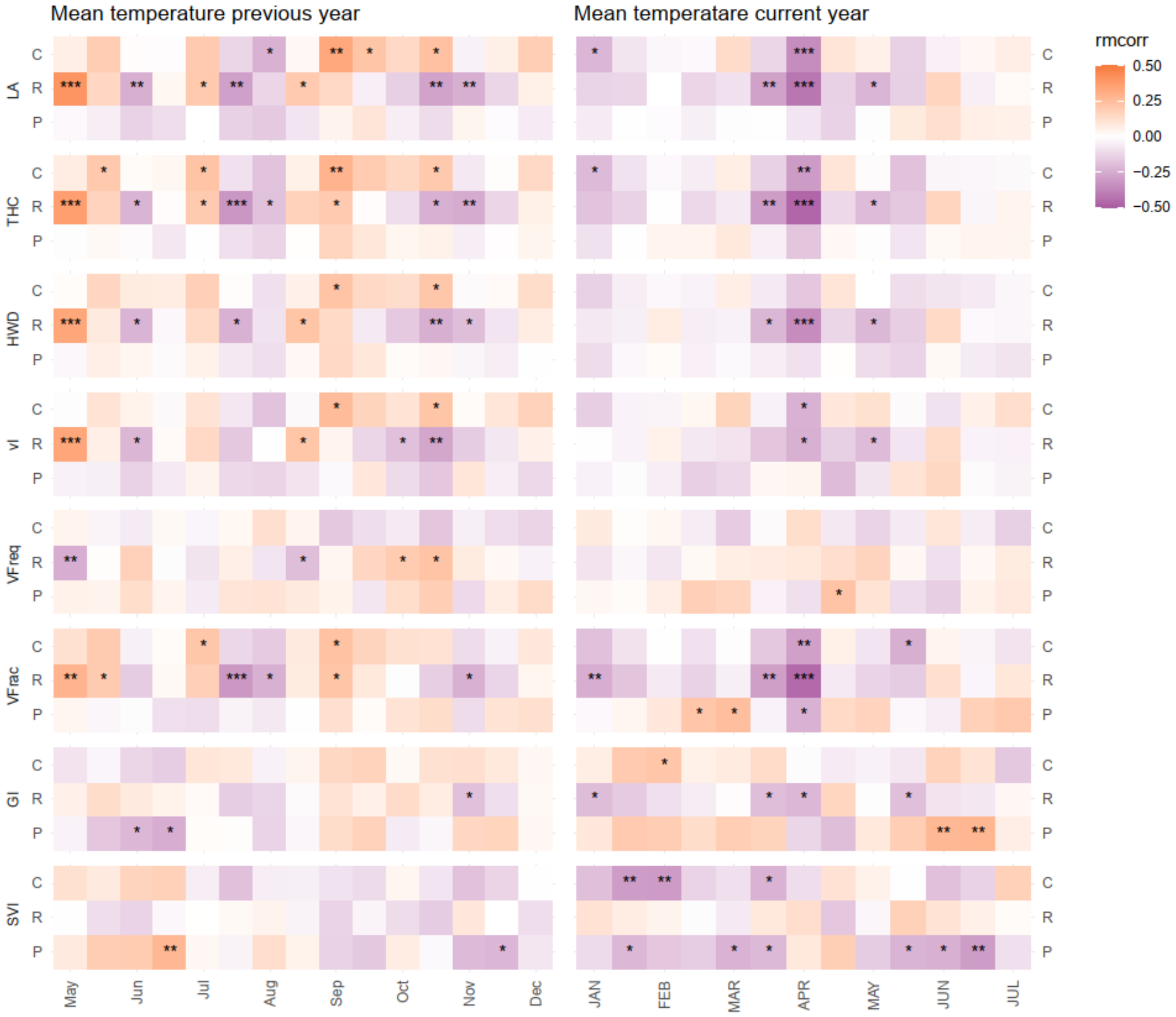

Fig. S5 Repeated measures correlation coefficients (rmcorr) between the detrended vessel traits (LA=mean vessel lumen areas, THC=theoretical hydraulic conductivity, HWD=hydraulically weighted diameter, VI=vulnerability index, VFreq=vessel frequency, VFrac=vessel fraction, GI=vessel grouping index, SVI=solitary vessel index) and detrended mean temperature of 30-day periods moved in 15-day steps from May to December of previous year and from January to July of the current year corresponding to the growth ring. The annotations for correlation significance *p<0.05, **p<0.01, ***p<0.001. P=park, R=residential street, C=central urban street

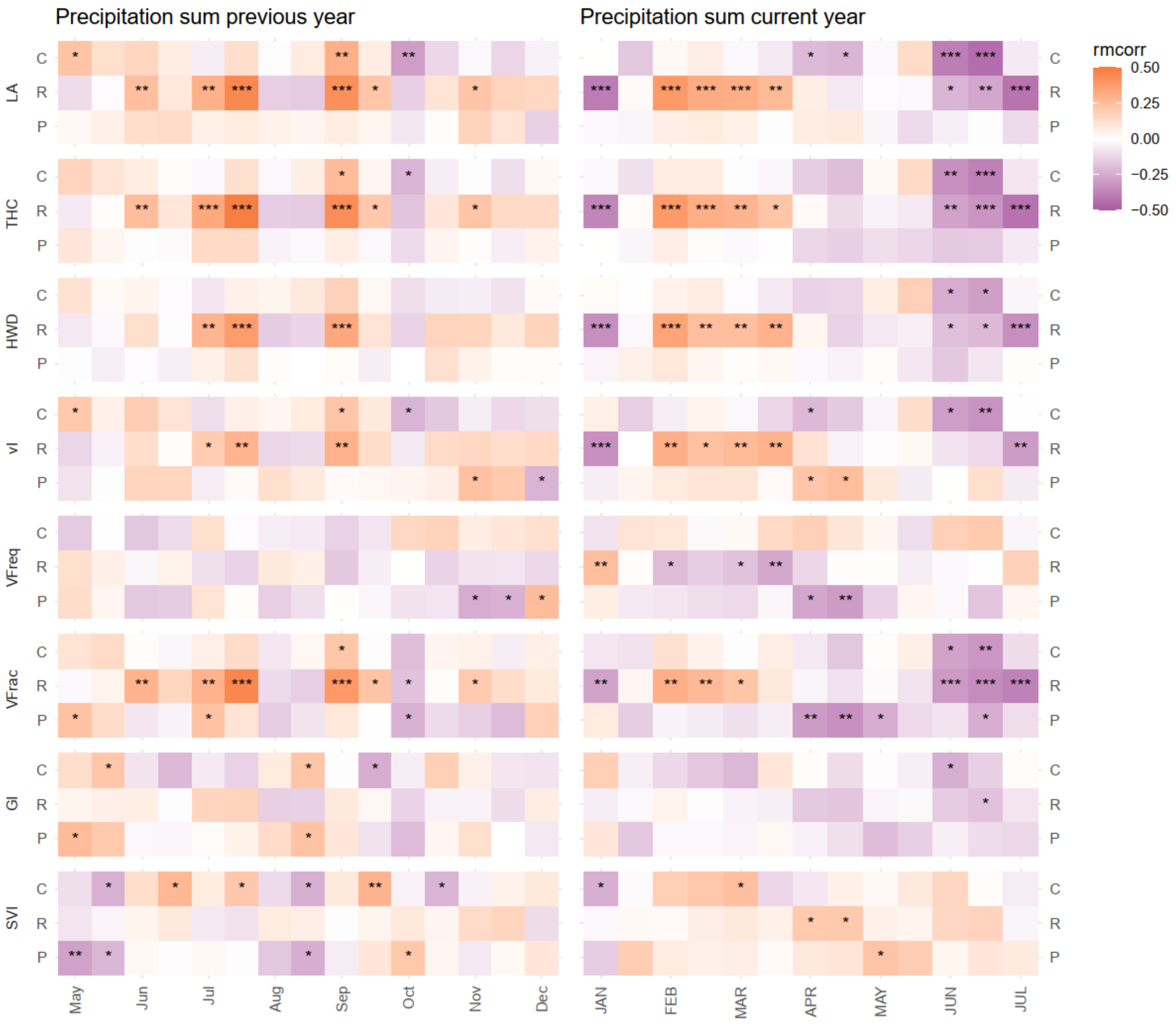

Fig. S6 Repeated measures correlation coefficients (rmcorr) between the detrended vessel traits (LA=mean vessel lumen areas, THC=theoretical hydraulic conductivity, HWD=hydraulically weighted diameter, VI=vulnerability index, VFreq=vessel frequency, VFrac=vessel fraction, GI=vessel grouping index, SVI=solitary vessel index) and detrended precipitation sum of 30-day periods moved in 15-day steps from May to December of previous year and from January to July of the current year corresponding to the growth ring. The annotations for correlation significance *p<0.05, **p<0.01, ***p<0.001. P=park, R=residential street, C=central urban street

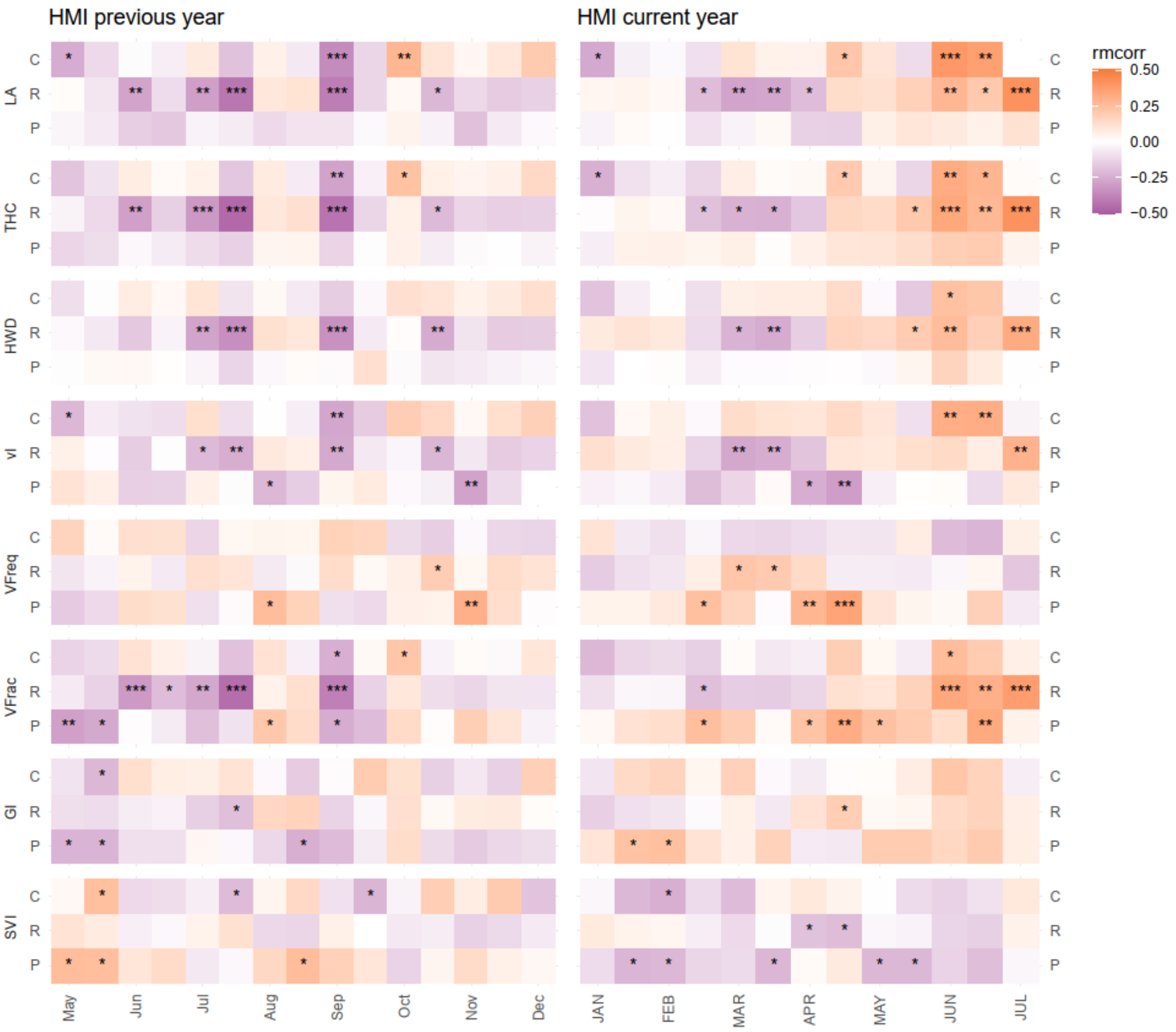

Fig. S7 Repeated measures correlation coefficients (rmcorr) between the detrended vessel traits (LA=mean vessel lumen areas, THC=theoretical hydraulic conductivity, HWD=hydraulically weighted diameter, VI=vulnerability index, VFreq=vessel frequency, VFrac=vessel fraction, GI=vessel grouping index, SVI=solitary vessel index) and detrended heat moisture index (HMI) of 30-day periods moved in 15-day steps from May to December of previous year and from January to July of the current year corresponding to the growth ring. The annotations for correlation significance *p<0.05, **p<0.01, ***p<0.001. P=park, R=residential street, C=central urban street
